# Trial of Martin the Incendiary

**Published:** 1829-07-01

**Authors:** 


### XXXV.

I. REPORT OF THE TRIAL OF JONATHAN MARTIN FOR SETTING FIRE TO THE YORK MINSTER. London, 8vo. pp. 92.

II. THE LIFE OF JONATHAN MARTIN, OF DARLINGTON, TANNER, WRITTEN BY HIMSELF. 8vo., pp. 50, 3d Edit. Lincoln, 1828.

The destruction of the venerable Cathedral of York, by fire, is fresh in the memory of all our readers, and the protracted trial of the incendiary excited considerable interest in the public mind. The initiatory examinations of the supposed culprit—and, we admit, the actual perpetrator, left not a doubt in the mind of every medical man of any experience or knowledge in his profession, that Martin was as decided a monomaniac as ever entered the walls of Bedlam. If there was no other testimony than the second publication at the head of this article, all doubt would vanish. The life of Martin, written by himself, is really a curiosity, and contains in every page—we might say in every line, intrinsic proof of disordered intellect. We are glad to observe, by reference to the report of the trial, that the professional witnesses, Mr. Caleb Williams, and Dr. Baldwyn Wake, of York, proffered a chain of evidence, under a close, and sometimes a galling cross-examination, which does them infinite credit. We cannot better express our entire approbation of the sentiments and conclusions contained in the evidences of Mr. Williams and Dr. Baldwyn Wake, than by giving them a permanent record in our Journal. It is a small but an honourable prize conscientiously adjudicated to merit. Would that we had more frequent occasions for expressing our approbation of medical evidence in courts of justice !

"Caleb Williams, on his affirmation. —*Examined by Mr. Sergeant Jones.*

Q. You are a surgeon in the city of York, I believe ?—A. Yes.

Q. Are you one of the medical attendants at the Lunatic Asylum, called the Friends' Retreat ?—A. Yes.

Q. Have you been conversant on the subject of the diseases of lunatics ?—A. Yes, for five or six years.

Q. When did you first of all see the prisoner at the bar, Martin, so as to observe his state of mind ?

A. On the 21st of the present month.

Q. How often have you seen him since that time ?

A. Daily, with the exception of two days.

Q. In the first place, I would ask you, what appears to you to be the state of his mind?—A. I consider him to be what is denominated a "*monomaniac*."

*Baron Hullock.* Just state, for the information of the jury, what that is.

*Witness.* It is a species of insanity, in which the delusion is confined to one idea only, or to one train of ideas.

Q. Is that the expression, that is well known and commonly used in medical treatises and discussions ?—A. Yes.

Q. It is opposed to delirium or delusion, upon all subjects generally ?

A. Yes.

Q. Then first of all I ask you, whether you have taken pains to satisfy yourself, whether the symptoms or appearances of his insanity are feigned or bona fide ?

A. I believe they are bona fide really.

Q. Have you had any discourse with him on the subject of dreams ?—A. I have.

Q. What is your impression with regard to the influence of dreams upon the mind of the prisoner ?

A. I believe his dreams to have more influence over him than they would have over a person of sound mind.

Q. To what subject do the dreams of the prisoner apply ?

A. Chiefly to the subject of religion.

Q. Did you also attend to his bodily strength ?—A. I did.

Q. Are there certain symptoms connected with his bodily strength, that afford any influence with regard to the state of his mind ?—A. There are.

Q. Did the appearances which you witnessed with regard to the state of his body, concur or run differently with his state of mind ; what is your opinion with regard to the state of his mind, as derived from those appearances ?

A. They confirmed my opinion with regard to the state of his mind.

Q. How was the eye ?—A. The eye was red.

Q. I need hardly ask you, whether persons acquainted with medical subjects of this sort, look to the eye of the patient as a test of their state of mind ?

A. The eye is frequently observed certainly, and the countenance generally.

Q. How was he as to his pulse ?—A. It was full, hard, strong, and quicker than natural.

Q. Is that state of the pulse the one commonly found in instances of monomania ?—A. Yes, during the period of excitement it is common.

Q. Do you think him a person that it would be safe to trust to go abroad ?—

A. I think the instances that have been given prove that it is not safe for him to go abroad.

Q. Then you are of opinion that he is insane ?—A. Yes.

Q. And that his madness is upon one particular subject, or upon one train of ideas ?—A. Yes.

Q. Are there many patients or persons in confinement who are labouring under monomania, and who have no other complaint ?—A. Yes, there are many ; I have opportunities of seeing many every week.

*Baron Hullock.* Were it otherwise he would not be denominated a monomaniac.

*Cross-examined by Mr. Alderson.*

Q. Are not those monomaniacs capable of distinguishing right from wrong ?

A. Not upon subjects upon which they are deluded.

Q. But upon general subjects?

A. Upon subjects perfectly unconnected with their delusion they have the power of judging of right from wrong.

Q. Upon those subjects upon which they are capable of so judging, they act like other persons capable of judging right from wrong?—A. Yes, frequently.

Q. Do they avoid dangers consequent upon their actions?

A. Frequently.

Q. Do they run away after having done a thing that they know is wrong?

A. Very commonly.

Q. Do they adopt skilful means to avoid the consequence of such an act?

A. They are very cunning in escaping punishment.

Q. And also very cunning in pursuing the means to accomplish an act?

A. Yes, the same.

Q. You don't consider that that shews that they know, before hand, they are doing a wrong act?—A. No.

Q. In what way would they shew they were doing a wrong act: would they do it by staying, if they did know they had done a wrong act. In what way do you, as a medical man, expect that insane persons would shew it?—A. I should think that an insane man would endeavour to avoid being detected.

Q. Then it is possible for a person, being an insane man, to do an act which he knows will be followed by punishment, and consequently endeavour to avoid it?—A. It is.

Q. Does not the fear of punishment deter them?

A. I believe the mind is so absorbed with the delusive idea, that no other idea occupies their attention.

Q. Do you not know, that persons who are insane are prevented doing such acts through fear of punishment?—A. No, that will not operate so as to prevent the delusion coming on.

Q. What, in your judgment, is the peculiar subject upon which this person, the prisoner, is to be looked upon as being mad?—A. An undue reliance upon his dreams, believing they are revelations of the divine will, directing him to perform certain acts.

Q. Has he told you that that is so?—A. Yes.

Q. Have you any other reason for knowing it, than that he has told you so?—A. No.

Q. Had you any other conversations with him before?—A. No.

Q. When did you first see him?

A. On the 21st of this month.

Q. That was the first day of the assizes?—A. Yes.

Q. All your interviews have been with him since the Judges came to town?—A. Yes.

Q. Since that, he has been telling you of his dreams?—A. Yes.

*Re-examined by Mr. Brougham.*

Q. You say you have had considerable experience in regard to the diseases of insane persons, from your attending a Lunatic Asylum?—A. Yes.

Q. Do you consider, judging from your experience, that the conduct and appearance of this prisoner could have been put on with the view of deceiving you, or that it was otherwise than real?

A. I don't think it could.

DR. BALDWIN WAKE, SWORN.—*Examined by Mr. Brougham.*

Q. You are a physician in York?—A. Yes.

Q. Have you had experience of lunatic patients?—A. Yes.

Q. Are you physician to a lunatic establishment?—A. Yes, I am; to the York Lunatic Asylum.

Q. How long have you been so?

A. For thirteen years and upwards.

Q. Have you seen the prisoner lately?

A. Since the 21st of this month, and also about three or four weeks ago, without his being aware that I did see him. I looked through the window of Mr. Kilby's apartment.

Q. Have you paid attention to his case lately?

A. Yes, I devoted an hour or two at a time, and investigated his case as carefully as time would allow me.

Q. During the visits you have made, and you have seen him almost daily, you have investigated his case?

A. Almost daily; and I have seen him occasionally twice a day.

Q. Now, judging from what you have seen of him, and applying the result of your own experience in such diseases to him, is it your opinion that he is of sound or unsound mind?—A. Of unsound mind, most undoubtedly.

Q. Of what description is the insanity he labours under.—A. *Monomania.*

Q. Is that a frequent species of the complaint?

A. Very frequent; there are a great many varieties.

Q. But the characteristic of the whole of them is, that of being under a delusion on one particular point, and more or less sane upon other points?

A. Yes, sir; that they have sanity upon most other points.

Q. Did you observe any particular bodily infirmity about him?—A. Yes.

Q. Had you occasion to observe his head?—A. I had.

Q. What did you observe in regard to it.

A. I observed that it has the appearance, on the frontal bone, as if he had had a wound or accident, and upon enquiring as to it, he told me a long detail of his burning the Minster, nearly as has been stated before; and in doing so, I observed that he suddenly put his hand to his head, and seemed quite lost for some time; that attracted my attention more particularly towards his head, and when he was apparently sufficiently recovered to answer questions—I asked him what was the matter, and he said, "when I talk much, a pain strikes me at that place where I had received a wound."

Q. Did you, in consequence of that observation, examine his head more particularly?—A. Yes, I did.

Q. What was the result of your examination?

A. I found there was the mark of an injury there, and I inquired into the particulars of it.

Q. I shall not trouble you to give a minute description of it, but you say there was there apparently the remains of an injury?—A. Yes.

Q. Did you feel the mark of that injury with your hand?—A. I did.

Q. What effect did the pressure of your hand make upon it?

A. Not much effect; but I found that there seem'd to be some little depression upon that part, and I inquired something further about it.

Q. I shall not ask you what he told you; did you make any inquiry as to his appetite?

A. I did, and I found that he had a most voracious appetite. I went down in the cell when his wife came to him with his dinner, and I observed that he eat in the most voracious manner a beef steak pie, and he devoured it in a short time, although it appeared to be as much as any three men could have eaten.

Q. Is that a symptom frequently attending the species of insanity you describe?

A. It is an attendant symptom upon insanity generally, where the health is at the same time good. There is generally a voracious appetite, and he devours his food in a particular manner.

Q. Did you observe his eye?—A. I did.

Q. What appearance had that?

A. It was glassy, and when he was excited, it betrayed a great deal of *maniacal* expression, or appearance in it, which would strike any person accustomed to those diseased patients, so that they cannot mistake the nature of their complaint.

Q. Could those bodily symptoms that you describe, have been put on for the purpose of deceiving you?—A. I presume not.

Q. Don't you believe they could not?

A. I do firmly believe they could not; it is a mistake when I use the other expression, that I presumed it could not be assumed.

Q. Had the pulse any thing to do with discovering the state of the patient?—A. The pulse was uncommonly hard, and not very frequent. I examined the state of the heart, but I found the action of the heart did not account for the strong pulse, I therefore attributed it to his delusion, and to the state of his mind. That symptom is frequently attendant upon insanity, when there is a restlessness and excitement.

Q. Is an inability to remain at rest, one of the symptoms?

A. I observed him when he did not know it, and perceived him walk up and down, apparently five or six miles an hour; and that was his usual manner.

Q. Was that observed frequently?—A. Yes.

Q. From the opportunities you had of seeing him, upon those occasions you speak of, have you any doubt that he is a person of unsound mind?—A. I have no doubt of it.

Q. Is he a person capable of distinguishing right from wrong?

A. Upon the subject of his delusion, I think not. I could state my reason for drawing that conclusion, if necessary, from another case that bears upon this one.

Q. No, I don't ask you that, but I may ask you, if a person, who has a delusion upon one particular subject, and is, upon that subject, incapable of distinguishing right from wrong, be capable, upon other subjects, of distinguishing right from wrong?

A. Generally, they can do so upon other subjects.

Q. Could you, upon other subjects, rely, even with certainty, on their distinguishing right from wrong ?—A. Yes.

Q. Upon subjects generally, excluding those to which this delusion applies, I ask you, are insane persons under the influence of fear ?—A. I think upon maniacs in general, fear operates very powerfully.

Q. And will they endeavour to effect their escape, after they have done a wrong act ?—A. Yes, they will endeavour to elude punishment.

Q. May a person labouring under this species of disease, called "monomania," be incapable of distinguishing right from wrong, and then doing that which we should call wrong, be able afterwards to know that they are likely to be punished for it ?

A. I think that when that delusion is frequently excited, a maniac does not know right from wrong; but then, after he has committed a crime, he may become sensible of his danger. I have known many instances of monomania, and I know that at certain times they have lucid intervals, and will be conscious of the error they have committed, and the delusion under which they labour; of this I know a striking case, if it were necessary upon this occasion to state it.

Baron Hullock summed up in a long, elaborate, and able manner. The fact of Martin's having set fire to the Minster was as clear as the sun at noon day. But the question of culpability—that is to say, of sanity or insanity, rested entirely, or almost entirely, on the medical witnesses. The jury instantly acquitted him, on the plea of insanity—and every man of the slightest medical information, must acquiesce in the decision. We tender our thanks and the profession of sincere esteem to Dr. Baldwin Wake and Mr. Williams.

---

